# Worldwide Population Dynamics of *Salmonella* Saintpaul: Outbreaks, Epidemiology, and Genome Structure

**DOI:** 10.3390/genes16030254

**Published:** 2025-02-22

**Authors:** Pedro Panzenhagen, Devendra H. Shah, Dalia dos Prazeres Rodrigues, Carlos Adam Conte Junior

**Affiliations:** 1 Center for Food Analysis (NAL), Technological Development Support Laboratory (LADETEC), Federal University of Rio de Janeiro (UFRJ), Cidade Universitária, Rio de Janeiro 21941-598, RJ, Brazil; conte@iq.ufrj.br; 2 National Reference Laboratory for Diagnosis of Enteric Bacteria (LABENT/LRNEB), Oswaldo Cruz Foundation, Av. Brasil, 4365-Manguinhos, Rio de Janeiro 21040-900, RJ, Brazil; daliarodrigues@yahoo.com.br; 3 Analytical and Molecular Laboratorial Center (CLAn), Institute of Chemistry (IQ), Federal University of Rio de Janeiro (UFRJ), Cidade Universitária, Rio de Janeiro 21941-909, RJ, Brazil; 4 Laboratory of Advanced Analysis in Biochemistry and Molecular Biology (LAABBM), Department of Biochemistry, Federal University of Rio de Janeiro (UFRJ), Cidade Universitária, Rio de Janeiro 21941-909, RJ, Brazil; 5 School of Veterinary Medicine, Texas Tech University, Amarillo, TX 79106, USA; devendra.shah@ttu.edu; 6 Graduate Program in Food Science (PPGCAL), Institute of Chemistry (IQ), Federal University of Rio de Janeiro (UFRJ), Cidade Universitária, Rio de Janeiro 21941-909, RJ, Brazil; 7 Graduate Program in Chemistry (PGQu), Institute of Chemistry (IQ), Federal Universit of Rio de Janeiro (UFRJ), Cidade Universitária, Rio de Janeiro 21941-909, RJ, Brazil; 8 Graduate Program in Biochemistry (PPGBq), Institute of Chemistry (IQ), Federal University of Rio de Janeiro (UFRJ), Cidade Universitária, Rio de Janeiro 21941-909, RJ, Brazil

**Keywords:** motility, intramacrophage survival, bacterial virulence, phenotypic characteristics, antimicrobial resistance, plasmid, MLST, WGS

## Abstract

Background/Objectives: *Salmonella* Saintpaul (SSa) is increasingly linked to foodborne outbreaks in Brazil and globally. Despite its rising public health significance, its epidemiology, genomic diversity, and pathogenic potential remain underexplored. This study addresses these gaps through a comprehensive global analysis of SSa population dynamics, outbreak patterns, and genetic structures, along with an in-depth phenotypic and genomic characterization of strain PP_BR059, isolated from a hospitalized patient in Ceará, Brazil. Methods: We analyzed 1,953 publicly available SSa genomes using core-genome multi-locus sequence typing (cgMLST), antimicrobial resistance (AMR) profiling, pan-genome analysis, and phylogenetic inference. A genome-wide association study (GWAS) identified genetic determinants of virulence and AMR. The invasiveness and intracellular survival of PP_BR059 were assessed using in vitro macrophage infection assays, while whole-genome sequencing (WGS) provided genetic insights. Results: Phylogenetic analysis identified 49 sequence types (STs), with ST-50 (787 genomes) and ST-27 (634 genomes) being most prevalent. ST-50 included all clinical strains from South America, including PP_BR059. AMR analysis showed 60% of SSa genomes were pan-susceptible, while ST-27 had the highest proportion of AMR strains. GWAS revealed distinct evolutionary lineages within ST-50 and ST-27. PP_BR059 exhibited lower macrophage invasion (3.82%) but significantly higher intracellular survival at 2 h (68.72%) and 20 h (25.68%) post-infection. WGS confirmed a pan-susceptible AMR profile and plasmid absence. Conclusions: This study highlights SSa’s global dissemination, evolutionary trends, and pathogenic variability, emphasizing the need for molecular surveillance to inform public health interventions.

## 1. Introduction

Non-typhoidal *Salmonella* (NTS) is a leading cause of bacterial foodborne illnesses worldwide [1]. NTS poses a significant public health threat and imposes a substantial economic burden for both developed and developing nations [2]. Among over 1400 known NTS serovars, *Salmonella* Saintpaul (SSa) ranks as one of the top ten most frequently detected serovars in humans and animals globally, accounting for approximately 1.6% of severe *Salmonella* infections [3]. In the United States, SSa has caused notable outbreaks, including those in 2008, 2009, and 2013, linked to contaminated peppers, alfalfa sprouts, and cucumbers, primarily sourced from Mexico [4,5,6,7,8]. For instance, the 2008 outbreak affected 1442 individuals across 43 states and D.C., leading to 286 hospitalizations and two fatalities. Initially traced to tomatoes, the outbreak strain was later linked to jalapeno peppers from various locations, including a farm in Mexico [9]. In the 2009 outbreak, alfalfa sprouts contaminated with SSa sickened 235 people in 14 states, with a hospitalization rate of 3% [4]; the Food and Drug Administration (FDA) traced the source to multiple sprout production facilities. The 2013 cucumber outbreak affected 84 people across 18 states, resulting in 17 hospitalizations [10], with the contamination traced back to farms in Mexico.

In Brazil, SSa is among the top 10 most prevalent *Salmonella* serovars, with 1222 cases (0.16% of 62,030 isolates) reported between 2011 and 2020 [11].These isolates originated from a variety of sources, including food (57%), the environment (16%), animals (14%), raw materials (8%), and humans (5%). While large-scale outbreaks of SSa have not been reported in Brazil, localized cases have been identified in specific neighborhoods or cities. SSa is also one of the top ten most frequently isolated *Salmonella* serovars from human sources in Brazil [11]. Recent studies have documented SSa isolation in the country [12,13,14]; however, despite extensive epidemiological data on its prevalence, there is a lack of comprehensive studies consolidating genetic data from sequenced SSa genomes associated with foodborne outbreaks. Furthermore, research on the invasiveness and virulence potential of clinical SSa strains is still limited.

This study aims to fill this gap by analyzing the global population dynamics of SSa, focusing on major outbreaks, epidemiological patterns, and genomic structures. Through this multidimensional approach, we seek to identify evolutionary trends, genetic determinants, and potential resistance and virulence factors that contribute to the pathogenicity and spread of SSa. Additionally, we provide a detailed phenotypic and genotypic characterization of the clinical SSa strain (PP_BR059) isolated from a hospitalized patient in the Ceará outbreak in northeast Brazil, specifically addressing its invasiveness and virulence potential in the context of human infection, which is similar to that of other NTS serovars.

## 2. Materials and Methods

### 2.1. Phenotypic Characterization of SSa Strain PP_BR059

Strain PP_BR059 is a clinical isolate of SSa obtained from the fecal sample of a patient hospitalized during a 2011 outbreak in Ceará, Brazil. The infection was characterized by severe symptoms, such as diarrhea and high fever, raising concerns about its potentially enhanced virulence and invasiveness compared to typical NTS strains. The strain was sent to the National Reference Laboratory for serotyping analysis and was included in the Enterobacteria Collection at the Oswaldo Cruz Foundation’s National Reference Laboratory. Later, PP_BR059 served as a critical model for this research, studying the virulence and pathogenicity of SSa, contributing to our understanding of its clinical severity, and aiding in the development of public health strategies. The strain was preserved in 15% (*v*/*v*) glycerol at −80 °C and cultured in either Brain Heart Infusion (Difco, USA) or LB broth Lennox (LB) medium for 16 h at 37 °C. For the experiments, a single colony was inoculated into 5 mL of LB medium and incubated overnight (16 h) at 37 °C, with shaking at 200 rpm.

Antimicrobial susceptibility testing of the SSa strain PP_BR059 was performed using the disk diffusion method on Mueller–Hinton agar (Difco, Becton Dickinson, MD, USA), according to the guidelines provided by the Clinical and Laboratory Standards Institute [15]. The following antibiotic disks (BD, Becton Dickinson, MD, USA) were utilized: ampicillin—10 μg (Am), amoxicillin/clavulanic acid—20/10 μg (Amc), amikacin—30 μg (An), chloramphenicol—30 μg (C), ciprofloxacin—5 μg (Cip), sulfisoxazole—0.25 μg (G), gentamicin—10 μg (Gm), nalidixic acid—30 μg (Nal), streptomycin—10 μg (S), sulfamethoxazole–trimethoprim—23.75/1.25 μg (Sxt), tetracycline—30 μg (Te), and ceftiofur—30 μg (XNL). The control strains included *Escherichia coli* ATCC 25922, *Staphylococcus aureus* ATCC 25923, and *Pseudomonas aeruginosa* 10536.

The motility of SSa strain PP_BR059 was determined following a protocol described previously [16]. Briefly, the LB medium supplemented with 0.3% (*w*/*v*) motility agar (Difco, USA) was used. A 2 μL suspension from overnight cultures of the strain PP_BR059 and reference strains *S.* Typhimurium SL1344 (ST19) and LT2 (ST19) were spot-inoculated onto the surface of the semisolid medium. The plates were then incubated at 37 °C for 7 h, after which the diameter of the growth halo was measured using a ruler. Each strain was tested in triplicate and the experiment was repeated independently three times. To analyze the data, the percentage of motility for the strain PP_BR059 was calculated relative to the reference strains, which were set as 100%. Statistical differences in motility between the strains and the reference were determined using one-way analysis of variance (ANOVA) with Dunnett’s post hoc test (*p* < 0.05).

The intra-macrophage survival of SSa strain PP_BR059 was determined following a previously described protocol [17]. Human macrophages (THP-1 cells) were seeded into a well plate with complete RPMI 1640 medium (HyClone Laboratories, Cytiva, Utah, USA) and activated by adding 10 mM phorbol 12-myristate 13-acetate (PMA) for 18 h at 37 °C with 5% CO_2_. An aliquot from the overnight culture was then transferred to an LB medium and incubated at 37 °C for 3 h before cell infection. The activated THP-1 cells were infected with a bacteria-to-cell ratio of 50:1 (bacteria:cell). To facilitate contact between the bacteria and cells, the plates were centrifuged for 10 min at 400× *g* at room temperature. After a 30 min incubation at 37 °C in a 5% CO_2_ incubator, the cells were washed thrice with phosphate-buffered saline (PBS). Subsequently, each well received an RPMI 1640 medium containing 200 μg/mL gentamicin. The cells were lysed at 30 min (for uptake/invasion), 2 h, and 20 h (for survival) of incubation using 200 μL of 0.5% Triton X-100 (Fisher Biotech, Waltham, MA, USA). Serial 10-fold dilutions were plated on LB agar to determine viable counts. Each isolate was tested in triplicate in three independent experiments. The percentage of intracellular counts was calculated as follows: the colony-forming unit (CFU) at t = 0 divided by the CFU at t = 30 min multiplied by 100 (for % uptake/invasion); the CFU at t = 30 min divided by the CFU at t = 2 h multiplied by 100 (for % intracellular survival at 2 h); and the CFU at t = 2 h divided by the CFU at t = 20 h multiplied by 100 (for % intracellular survival at 20 h). Statistical differences (*p* < 0.05) in intracellular survival between the strains were determined using one-way analysis of variance (ANOVA) with Dunnett’s post hoc test, comparing the strains to the reference *S.* Typhimurium strains, LT2 and SL1344 (ST19).

### 2.2. WGS of SSa Strain PP_BR059

The genome of the SSa strain PP_BR059 was sequenced using the Illumina© MiSeq platform. Paired-end (2 × 250 bp) Nextera XT libraries (Illumina©, San Diego, CA, USA) were prepared following the manufacturer’s suggested protocol. DNA samples were multiplexed using Illumina-supplied barcodes; DNA pools were size-selected to be in the range of 600–1000 bp (with an average peak at ∼800 bp). The read quality was assessed using FastQC v0.11.5. Reads were processed with Trimmomatic 0.36 [18]; short reads were length-adjusted using FLASh v1.2.11 [19]. The final treated reads were assembled using SPAdes v3.11.1 [20]. Genome statistics were obtained using QUAST v4.6.0 [21]. The assembly of strain PP_BR059 generated 38 contigs, with 24 contigs greater than or equal to 300 bp. The assembly covered a total of 4687,206 bp, with an N50 value of 379,274 bp and an average read coverage of 188×. The average G + C content was 52.23%; the genomic features, including genes, CDS, and transcripts, numbered 4604. The whole-genome assembly of strain PP_BR059 has been deposited in the NCBI GenBank under the BioSample number SAMN05505497. The raw reads can be found in the Single Read Archive (SRA) run SRR4046393; the assembled contigs are available under the accession number GCA_009537095.1.

### 2.3. Comparative Genomic Analysis of Salmonella Saintpaul

A total of 1953 assembled genomes of SSa strains were obtained from the Pathogen Detection Web Browser on the National Center for Biotechnology Information (NCBI) website “https://www.ncbi.nlm.nih.gov/pathogens/ (accessed on 30 August 2020). These records were retrieved by using the search keyword “*Salmonella* Saintpaul” and were downloaded for all available assemblies as of 30 August 2020 (Supplementary Table S1). The genomes consist of isolates collected from 16 countries spanning North America, Latin America, Asia, Africa, Europe, and Oceania. These isolates were sourced from diverse origins, including the environment, food, animal waste, human feces, and blood. The isolation dates of the samples range over 30 years, from 1988 to 2020. The quality of the selected genomes was assessed using STARAMR [22] with the following three exclusion criteria: (1) a final genome size of less than 4 Mb or greater than 6 Mb; (2) an N50 value exceeding 10 kb; and (3) at least one contig equal to or greater than 300 bp. All follow-up in silico genomics analyses were conducted using the sequenced genome of SSa strain PP_BR059 and 1953 SSa genomes downloaded from a publicly available database.

Core-genome multi-locus sequence typing (cgMLST) was performed using cgMLSTFinder. V1.2. The cgMLSTFinder utilizes a rapid and accurate K-mer alignment of the reads [23] against EnteroBase to identify the cgMLST schemes based on alleles found in the 3002 *Salmonella* genes [24]. The traditional MLST was performed using PubMLST (https://pubmlst.org/organisms/salmonella-spp, accessed on 30 August 2020), which employs WGS data to identify the sequence types (STs) of bacteria [25].

The in silico antimicrobial resistance (AMR) gene and plasmid detection was conducted using STARAMR 0.7.1 with the following settings: a minimum DNA identity threshold of 95%; and a minimum DNA coverage of 60% for all genome alignments [22]. STARAMR scans bacterial genome contigs against the ResFinder [26], PointFinder [27], and PlasmidFinder [28] databases, provides a summary report of the detected AMR genes and plasmids, and predicts drug resistance based on the identified resistance genes.

Pan-genome analysis and core-genome phylogenetic inference were conducted using Roary, v1.14.5 [29]. The pan-genome analysis and core-genome alignment were performed with a minimum percentage identity of 95% for blastp; a gene was required to be present in at least 99% of the isolates to be considered as core. To ensure standardized genome annotation, all genomes were annotated using prokka, v1.14.5 [30]. The pan-genome visualization was achieved by employing the postprocessing scripts provided by Roary. For the generation of a maximum likelihood phylogeny from the core-genome alignment, IQtree v2.0.3 [31] was utilized, employing a GTR + F + I + G4 substitution model with 1000 bootstrap replications to support the tree nodes. Complementary annotation was performed using emapper, v2.0 [32] with default parameters, relying on eggNOG orthology data [33]. Diamond protein alignment [34] was employed for the sequence searches. For interactive visualization of the phylogenetics, metadata, and gene content, the browser application Phandango was used [35].

Detection of the WGS variants and the prediction of their effects were conducted using Snippy, v4.6.0 (Seemann T., GitHub repository: snippy). The complete genome sequence of SSa strain CFSAN004175 (GCA_001952995.1) served as the reference strain for identifying single-nucleotide variants (SNVs). To enhance the accuracy of phylogenetic reconstructions, recombinant regions within the core-genome alignment were identified and excluded using Gubbins v2.4.1 [36]. Phage regions and Ribosomal RNA were identified using Phastaf, v0.1.0 (Seemann T., GitHub repository: phastaf) and Barrnap, v0.9 (Seemann T., GitHub repository: barrnap), respectively, to facilitate masking in the reference genome. The final set of high-quality core-genome SNPs was extracted from the alignment file using SNP-sites, v2.5.1 [37]. Finally, IQtree was employed for the SNP phylogenetic inference, following previously described methods.

## 3. Results and Discussion

### 3.1. Comparative Genomic Analysis of SSa

**Figure 1:**
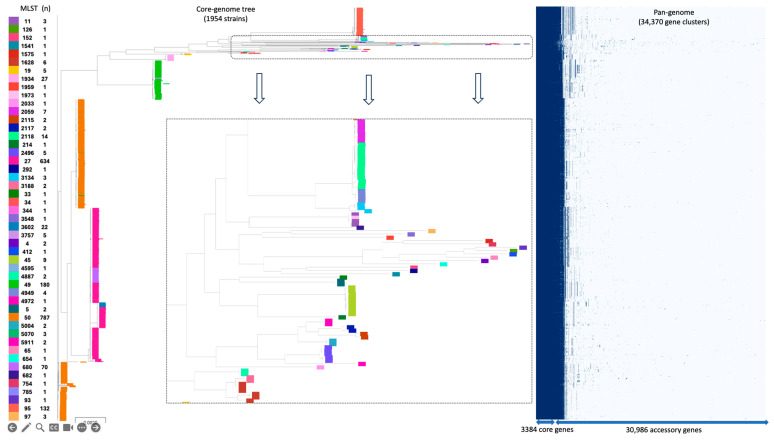
Phylogenetic tree based on the concatenated sequence alignment of core genes identified by Roary, showing the distribution of multi-locus sequence types (MLST) across 1954 *SSa* genomes, including the *PP_BR059* strain sequenced in this study. The corresponding pan-genome matrix on the right illustrates the distribution of core (dark blue) and accessory (light blue) genes across all analyzed genomes.

The MLST analysis identified a total of 49 distinct sequence types (STs) of SSa, with ST-50 being the most prevalent, encompassing 787 genomes, followed by ST-27, comprising 634 genomes (Figure 1). Together, these two STs accounted for 1421 (73%) of the 1954 SSa genomes analyzed in this study (Supplementary Table S1). The other most frequently detected STs included ST-49, ST-95, and ST-680, with 180, 132, and 70 genomes, respectively. The remaining STs had fewer than 30 representative genomes. Our analysis revealed that the strain PP_BR059 belongs to MLST 50 (ST-50). Given the significant role of SSa ST-50 in the genus *Salmonella* epidemiology globally, the high phenotypic virulence exhibited by the PP_BR059 strain underscores the importance of molecular epidemiology for investigating outbreaks, especially when dealing with relatively uncommon organisms and limited knowledge of their diversity and distribution.

Based on the provided metadata on ST-50 sources (Supplementary Table S1), around 100 non-clinical isolates originate from turkey and turkey products. The frequent isolation of ST-50 from turkey (e.g., “Young Turkey,” “Turkey Carcass Sponge,” “Turkey Meat”) in the United States [38] and Europe [39,40] suggests that poultry may be a significant risk factor for human SSa infections worldwide. This aligns with global findings that often implicate poultry and turkey as major sources of *Salmonella* contamination in the food supply chain [41]. Interestingly, environmental sources, such as canal water, river water, and food-processing residues also indicate potential indirect transmission routes (Supplementary Table S1). Additionally, regional distinctions, such as the higher reporting of turkey isolates in the United States and other animal sources in Brazil, may reflect differences in food production systems or surveillance practices.

Interestingly, most ST-50 genomes originated from three BioProjects (Supplementary Table S1). BioProject PRJNA230403 contained 279 SSa clinical strains (stool, urine, rectal swabs, tissues, wounds, and blood) sequenced by PulseNet USA at the Center for Disease Control and Prevention. BioProject PRJNA248792 included 202 SSa strains of clinical origin sequenced by the *Salmonella* Reference Service at the Gastrointestinal Bacteria Reference Unit in the United Kingdom. BioProject PRJNA596817 comprised 95 SSa strains originating from routine public health outbreak investigations in New South Wales (NSW), Australia [42]. In 2019, an outbreak of SSa ST-50 in Scotland involved person-to-person transmission and hospitalizations, primarily affecting children [43]. Although WGS was used to investigate the outbreak [43], the specific genomes identified in the study were not found in pathogen-detection databases, preventing their inclusion in the current analysis. According to the study, 28 isolates of SSa were isolated from patients in Scotland with no travel history between October 2017 and November 2018. The outbreak strain formed a distinct cluster compared to other isolates from Scotland during the same period; no links were found to international isolates within the Enterobase database (containing over 180,000 *Salmonella* genomes).

The SSa ST-50 has also been implicated in various global outbreaks, often associated with food contamination, raw fruits or vegetables, and water [7,44,45,46,47,48,49] including the 2008 multi-state foodborne outbreak in the United States that affected over 1400 individuals [7]. Finally, SSa strains CFSAN004173, CFSAN004174, and CFSAN004175, isolated from the 2013 multistate cucumber-associated outbreak in the United States, were also classified as ST-50 (Supplementary Table S1). The frequent association of SSa ST50 with outbreaks and isolation from diverse clinical sources strongly suggests that ST50 is likely the predominant type responsible for human SSa infections worldwide.

While most ST-50 genomes in the NCBI database were derived from human clinical sources, over half of the ST-27 genomes (331 out of 634) were sourced from ground turkey in the United States (Supplementary Table S1). These genomes, collected between 2004 and 2018, were part of twelve BioProjects that conducted WGS of SSa as part of multi-state outbreak surveillance routines in the United States (Supplementary Table S1). Furthermore, BioProject PRJNA248792 documented a significant population of ST-27 strains (164 out of 634) originating from human clinical sources in the United Kingdom, recorded from 2013 to 2020. Similarly, BioProject PRJNA230403 included data on 75 ST-27 and 64 ST-95 strains extracted from human stool samples in the United States. All ST-49 genomes contained in BioProject PRJNA248792 were sourced from human clinical cases in the United Kingdom. Lastly, ST-680 was also a highly prevalent sequence type (*n* = 70) within this SSa collection because it was frequently isolated from ground turkey multi-state outbreak surveillance in the United States (Supplementary Table S1).

In silico resistome analysis showed that SSa exhibits a predominantly pan-susceptible genetic profile with 60% (1149 out of 1954) of the genomes carrying no AMR genes. Pan-susceptibility frequencies were observed across the predominant sequence types as follows: ST-50 with 574 out of 797 (72%); ST-27 with 236 out of 634 (37%); ST-49 with 114 out of 180 (63%); ST-95 with 109 out of 132 (83%); and ST-680 with 37 out of 70 (53%). Collectively, pan-susceptibility in other STs accounted for 79 out of 151 (52%). These results highlight that while most predominant sequence types (STs) exhibit high proportions of pan-susceptible strains, ST-27 stands out, with only 37% of the strains being pan-susceptible. This lower frequency suggests that ST-27, predominantly isolated from turkeys, is more likely to carry resistance genes compared to other STs, indicating a higher potential for AMR within this lineage. A probable reason for this observation is the intense use of antimicrobials in the poultry production chain, which likely exerts a selective pressure that favors the persistence and proliferation of MDR strains in ST-27.

**Figure 2:**
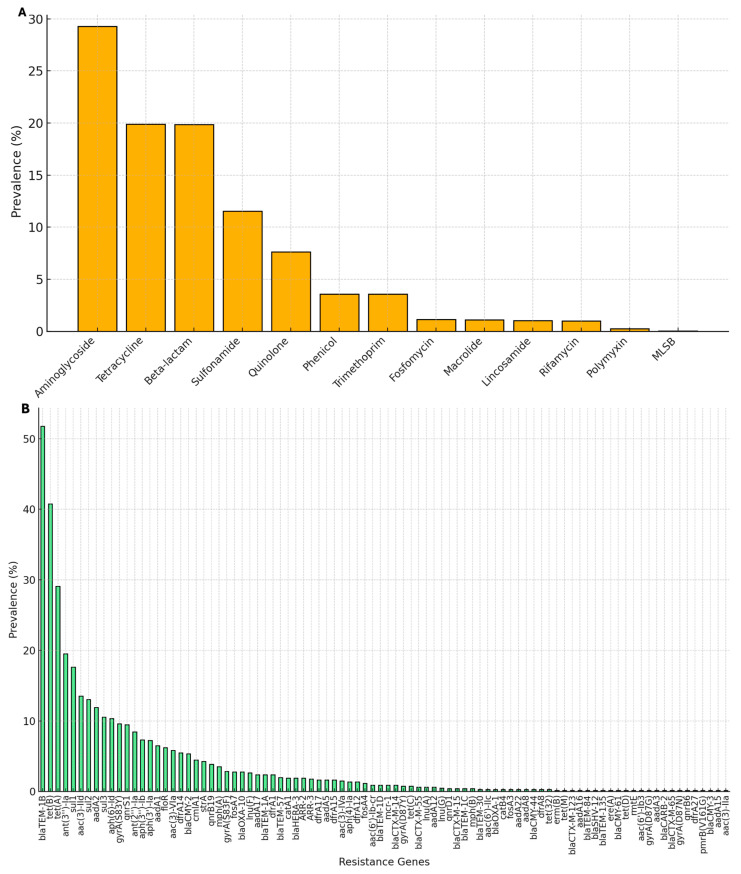
Prevalence of AMR genes in the 805 SSa strains: (**A**) distribution by antibiotic class; and (**B**) prevalence of individual resistance genes.

In contrast, the resistome of the 805 SSa strains, each carrying a minimum of one resistance gene, exhibits a rich diversity encompassing 90 distinct AMR genes (Figure 2 and Supplementary Table S1). Aminoglycosides were the most prevalent class, with resistance genes detected in approximately 30% of strains. This was followed by tetracyclines (19.9%) and β-lactams (19.9%), which also exhibited high prevalences. Conversely, rifamycin, polymyxins, and MLSB (macrolide–lincosamide–streptogramin B) showed significantly lower frequencies, each accounting for less than 2% of strains (Figure 2A). Regarding the resistance genes, *bla*TEM-1 (β-lactamase activity) and *tet* (A and B) (tetracycline resistance) emerge as the most frequently identified genes, present in over 50% of strains. Other highly prevalent genes include *ant* (3″)-Ia (aminoglycoside resistance) and *sul* (1 and 2) (sulfonamide). A long tail of less prevalent genes reflects the diversity and specificity of AMR gene occurrence among the strains with resistance genes that have emerged recently, conferring resistance to the last generation of antimicrobials, such as *bla*CTX-M, TEM- or SHV-type extended-spectrum ß-lactamases (ESBLs), CMY, and mcr, detected in SSa (Figure 2B and Supplementary Table S1). The great concern about the emergence of such resistance genes is their ability to encode resistance against last-resort drugs, such as ESBL, polymyxins, oxazolidinone and phenicol, in the treatment of multidrug-resistant (MDR) enterobacteria [50,51,52].

Out of the 1954 SSa strains evaluated in this study, 391 (20%) carried resistance genes indicative of an MDR phenotype; seven strains carried the *mcr-1* gene, and seventeen genomes carried the CTX-M gene (Supplementary Table S1). Interestingly, strains carrying these two resistance genes were isolated from different countries, including the United States, Mexico, Singapore, Australia, the United Kingdom, Thailand, Ethiopia, and Canada (Supplementary Table S1). SSa strains exhibiting MDR profiles have also been reported in various countries, including Morocco [53], Ethiopia [54,55,56], Denmark [40,57], Germany [39], Vietnam [58], Korea [59,60,61], and Brazil [14,62,63,64]. Interestingly, only a few reported outbreaks of SSa worldwide have been caused by MDR SSa strains [43,48,65]. Thirty-three percent of the MDR strains (130 out of 391) originated from the outbreak of *Salmonella* infections linked to raw turkey products in 2018 [38]. Additionally, the majority of other MDR genomes (*n* = 123) were deposited by the Gastrointestinal Bacteria Reference Unit in the United Kingdom and mainly derived from human clinical samples. It is worth noting that there is currently a high incidence of MDR strains within the entire genus *Salmonella* worldwide [66,67,68], particularly in developing countries with intensive animal production, such as Brazil, where the use of antimicrobials in livestock likely creates substantial selective pressure [69]. The reasons behind the lower frequency of AMR in SSa compared to other NTS *Salmonella* remain unexplained. Accurate determination of AMR is crucial in establishing national and global priorities, guiding public health interventions, and making treatment decisions regarding bacteria capable of causing infectious diseases or contributing to clinical infections, especially those associated with outbreaks.

The strain PP_BR059 also exhibited pan-susceptible phenotypic resistance. The results obtained from the software STARAMR showed agreement between genetic resistance, predicted phenotypic antimicrobial resistance, and phenotypic resistance observed in the disk diffusion test against a panel of 12 antimicrobials (Table 1). All downloaded genomes passed the quality check module of STARAMR (Supplementary Table S1). Interestingly, sixty-seven percent (1310 out of 1954) of the genomes, including the strain PP_BR059, showed no evidence of plasmids (Supplementary Table S1). The most frequently detected plasmids belonged to the incompatibility group I1 (IncI1), present in 369 out of the 1954 genomes (18.88%). Within this incompatibility group, we identified 110 out of the 1954 genomes (5.6%) that displayed no resistance genes, suggesting a pan-susceptible profile. On the other hand, 44 genomes carried IncI1 plasmids with the resistance genes *bla*CMY-2 and *bla*CTX-M, which confer resistance to the ESBLs ceftriaxone and cefoxitin. Although our analysis did not demonstrate that resistance genes are carried on the IncI1 plasmid, these plasmids are commonly detected in enteric bacteria and are also known to contribute to the horizontal transmission of AMR genes among enteric pathogens [13,70]. The combination of plasmids IncFIA (HI1), IncHI1A, and IncHI1B (R27) was the second most common, detected in 48 genomes; the IncX4 plasmid, the third most common, was present in 36 strains. Interestingly, all detected plasmid-mediated colistin resistance *mcr*-1 genes were found in the incompatibility group X4 plasmids (IncX4). Consistent with previous reports [71,72,73], IncX4, along with IncHI2 and IncI2, accounted for over 90% of reported *mcr*-1-bearing plasmids [74].

The pan-genome analysis of 1954 SSa genomes identified a total of 34,370 genes. Among these, 3384 genes were identified as core genes present in over 99% of the strains, while 30,986 genes were identified as accessory genes present in at least one strain, and 13,967 unique genes found in individual genomes (Figure 1 and Supplementary Table S2). Our pan-genome analysis aligns with previous studies on the pan-genome of *S*. enterica, based on relatively large datasets of genomes. For instance, Laing et al. identified a conserved core of 3200 genes, and a pan-genome of 25,300 genes from 4893 genomes [75]. Similarly, Park and Andam identified a conserved core of 2636 genes and a pan-genome of 33,257 genes from 297 genomes [76]. Finally, Yin et al. analyzed the genomes of 68 SSa strains and identified 3529 core genes in a pan-genome of 6360 genes [77]. The exact size of the *Salmonella* pan-genome depends on the total number of genomes analyzed and the chosen methodology [78]. However, it is evident that the pan-genome exhibits an “open” structure, similar to other pathogens such as *E. coli* [79]. Consistent with Yin’s study, the conserved core genome of SSa identified here has likely reached a stable minimum; any additional strains would not substantially affect the core-gene content (Figure 1). However, the increased frequency of detection of accessory genes suggests the accumulation of unique genes found only in a few or unique isolates. Most of these accessory genes are attributed to mobile genetic elements (MGEs) due to *Salmonella*’s propensity to acquire and lose genetic content for adaptation to different hosts.

The cgMLST analysis revealed extensive diversity within the SSa strains (Supplementary Table S1). The allelic differences in cgMLST resulted in significantly higher discriminatory power when compared with traditional MLST, classifying 1954 genomes into 1437 groups, whereas traditional MLST generated 49 groups (Figure 1 and Supplementary Table S1). In general, most identical cgMLST schemes were found among strains from the same geographic origin, indicating their potential involvement in the same reported outbreak (see geographic location column in Supplementary Table S1). However, we identified some highly similar strains (with identical cgMLST schemes) reported in multiple countries, suggesting the global transmission of SSa, possibly through infected travelers or contaminated foods (highlighted in yellow in Supplementary Table S1). For example, the strains FDA478976-2 and PNUSAS026954 were collected from Mexico in 2008 and the United States in 2017, respectively, from jalapeño peppers and human stool. They were likely involved in the 2008 multi-country and multi-state foodborne outbreak of SSa, which caused over 1400 illnesses in the United States [5,7,44]. Moreover, strains 37779 and FDA8656199-1 were isolated in 2014 from the United Kingdom and the United States, respectively, from human clinical sources and avocados (Supplementary Table S1). Given the multiple potential pathways of transmission, including international travel, imported foods, and animal movements, it is not possible to definitively link these strains to United Kingdom-wide outbreaks based solely on the provided metadata. Lastly, as part of routine public health outbreak investigations in NSW, Australia, between October 2015 and December 2018, 37 isolates belonging to the BioProject PRJNA596817 underwent WGS [42,80]. Interestingly, strain 244421 from the United Kingdom, isolated from a human clinical source, shared an identical cgMLST scheme with these 37 Australian isolates, increasing the likelihood of SSa’s potential involvement in the global dissemination of outbreak strains responsible for human clinical manifestations.

**Figure 3:**
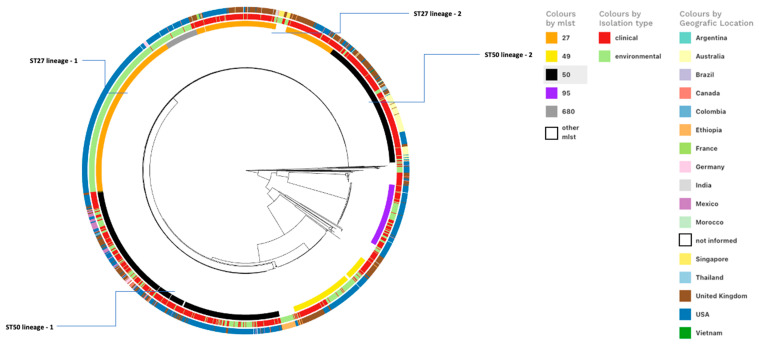
Genome-wide association analysis of single-nucleotide polymorphisms (SNPs) from the 1954 SSa strains, considering the top five most frequent sequence types, the geographic location, and the isolation type. The clinical isolation type is represented by strains originating from sources related to human and animal stools, urine, rectal swabs, tissues, wounds, and blood. The environmental isolation type is represented by strains originating from sources related to the environment, food, and water.

We conducted a genome-wide association study focusing on the top five most frequent sequence types (Figure 3). In this analysis, clinical isolates were categorized as strains from human and animal stools, urine, rectal swabs, tissues, wounds, and blood samples. In contrast, environmental isolates were represented by strains from environmental, food, and water sources. The SNPs analysis revealed that the two most prevalent sequence types, ST-50 and ST-27, diverged into two distinct lineages (Figure 3). ST-50 Lineage-1 consisted of 514 isolates, predominantly isolated from the United States (317), the United Kingdom (113), Mexico (48), and all strains from South American countries, including Brazil, Argentina, and Colombia (Supplementary Table S1). This lineage exhibited 37 unique SNPs and showed a balanced distribution between environmental and clinical sources. Conversely, ST-50 Lineage-2 comprised 277 isolates, primarily isolated from Australia (106), the United Kingdom (104), and the United States (57), along with strains from Thailand and Vietnam. Lineage-2 was characterized by the accumulation of 187 SNPs, with most isolates (253) originating from clinical sources. These findings suggest that Lineage-2 diverged from Lineage-1 through the acquisition of novel SNPs. It is plausible that these allelic differences have contributed to the persistence of these strains in clinical environments, as well as to their distinct geographic distribution, which is more prevalent in Europe, Asia, and Oceania compared to the Americas.

Regarding ST-27, Lineage-1 consisted of 377 isolates, primarily from the United States (349), and 29 other isolates from the United Kingdom, France, and Germany (Supplementary Table S1). This lineage displayed 82 unique SNPs and was mainly associated with environmental sources. In contrast, ST-27 Lineage-2 included 279 isolates, predominantly from the United Kingdom (*n* = 156) and the United States (*n* = 88), along with 35 strains from Australia, Canada, Ethiopia, and India. Lineage-2 was marked by 537 unique SNPs, with most isolates originating from clinical sources. Similar to ST-50, we hypothesize that ST-27 Lineage-1 and Lineage-2 are undergoing divergent evolutions via the accumulation of distinct SNPs, which are likely contributing to the differential niche adaptation in different geographic areas. Additionally, the ST680 cluster, comprising 70 strains, evolved from ST-27 Lineage-1 with a difference of only 6 unique SNPs. While most of these strains originated from environmental sources, six were derived from clinical samples.

These findings offer valuable insights into the phylogenetic relationships and evolutionary dynamics of SSa genomes. The accumulation of unique SNPs in Lineage-2 of both ST-50 and ST-27 suggests ongoing genetic changes that could influence the clinical behavior, geographic distribution, and environmental adaptation of these strains. Further research is needed to clarify the functional implications of these SNPs and their role in the epidemiology and pathogenicity of SSa. Gaining a deeper understanding of the evolutionary dynamics of these strains is crucial for developing effective strategies for the surveillance, prevention, and control of this pathogen and its associated diseases.

### 3.2. Invasiveness and Virulence Potential of the SSa Strain PP_BR059

**Figure 4:**
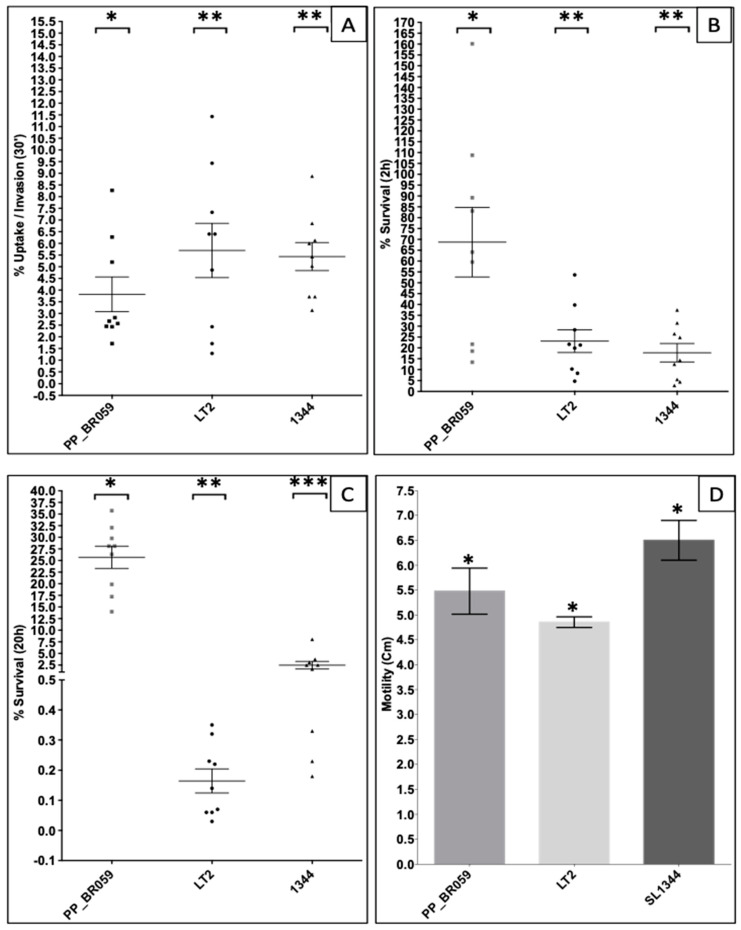
Percent uptake/invasion at 30 min (**A**). Survival at 2 h (**B**); and 20 h (**C**) post infection of the strain PP_BR059 in human macrophages (THP-1 cells). Motility of the strain PP_BR059 compared with the reference ST19 strains LT2 and SL1344 (**D**). Each strain number represents the mean from at least three independent experiments. Statistically significant (*p* < 0.05) differences are indicated by the number of asterisks (*/**/***).

To assess the virulence level and invasiveness of the PP_BR059 strain, we compared its invasion and intracellular survival in the human myelogenous leukemia macrophage (THP-1) cell line with the reference strains *Salmonella* Typhimurium LT2 and SL1344. Strain PP_BR059 exhibited a significantly low initial invasion/uptake (3.82%) in cultured human macrophages (Figure 4A); however, this strain showed significantly higher intramacrophage survival at 2 h (68.72%, Figure 4B) and 20 h (25.68%, Figure 4C) post infection, when compared with the reference strains LT2 (5.69%/23.10%/0.16%) and SL1344 (5.43%/17.77%/2.47%), respectively. *Salmonella enterica* serovars can cause systemic infections by invading non-phagocytic intestinal cells and can survive within phagocytic defense cells such as macrophages and dendritic cells [2,81,82,83,84,85,86]. SSa is typically associated with outbreaks of NTS gastroenteritis worldwide [4,5,6,7,8,9,39,45,48,57,87], although some cases of systemic infections have been reported [43,47,88]. Higher intra-macrophage survival, particularly after 20 h post phagocytosis, suggests that strain PP_BR059 is likely more invasive than other strains. Although strain PP_BR059 was isolated from a fecal sample of a case associated with an outbreak, the patient’s medical records were unavailable to confirm whether the infection became systemic. We recently reported NTS *Salmonella* Typhimurium in Brazil with high intramacrophage survival rates originating from systemic sites such as blood and cerebrospinal fluid [17]. It is currently unknown if SSa strains circulating in Brazil are also evolving to become more invasive.

Given that strain PP_BR059 exhibited significantly lower phagocytosis rates (Figure 4A), we hypothesized that this strain may also have lower motility compared to other NTS reference strains such as LT2 and SL1344 [89,90,91,92,93,94]. Surprisingly, strain PP_BR059 showed no impaired motility and did not significantly differ in motility compared to the reference strains (Figure 4D). These findings suggest that the ability of strain PP_BR059 to evade phagocytic engulfment is likely independent of the flagellar motility. To gain a better understanding of the genetic features underlying the above phenotypic characteristics, we performed WGS of strain PP_BR059. The genetic characterization of this strain is presented in the above section of this study.

## 4. Conclusions

In conclusion, this study provides valuable insights into the genomic epidemiology, antimicrobial resistance, and pathogenic potential of *Salmonella enterica* serovar SSa. Our analysis revealed substantial genomic diversity within SSa, with ST-50 and ST-27 accounting for the majority of global isolates. While ST-50 exhibited broad geographic distribution, ST-27 showed evidence of evolutionary divergence, with a subset associated with clinical infections and a higher prevalence of antimicrobial resistance genes. The characterization of strain PP_BR059, isolated from a hospitalized patient in Brazil identified this isolate as ST-50 and showed a significantly higher intra-macrophage survival rate at 2 h and 20 h post-infection. Whole-genome sequencing confirmed that PP_BR059 is pan-susceptible to antimicrobials, consistent with the overall trend of low resistance prevalence among SSa isolates from the global database. These findings underscore the need for continued genomic surveillance to monitor the evolutionary dynamics of emerging non-typhoidal *Salmonella* serovars. Understanding the genetic and phenotypic determinants of virulence and antimicrobial resistance in SSa will be crucial for assessing its public health significance and informing effective control strategies.

## Figures and Tables

**Table 1:** The panel of antimicrobial drugs used to determine the phenotypic AMR level of the Strain PP_BR059.

Antimicrobial Drug	Susceptible ^1^ (S)	Intermediary Resistance ^1^ (I)	Resistant ^1^ (R)	PP_BR059	*E. coli* ^2^	*S. Aureus* ^2^	*P. Aeruginosa* ^2^
Ampicillin (AM—10 µg)	≥17	14–16	≤13	24 (S)	19 (16–22)	31 (27–35)	R
Chloramphenicol (C—30 µg)	≥18	13–17	≤12	26 (S)	26 (21–27)	21 (19–26)	R
Gentamicin (GM—10 µg)	≥15	13–14	≤12	15 (S)	18 (19–26)	21 (19–27)	25 (16–21)
Amikacin (AN—30 µg)	≥17	15–16	≤14	22 (S)	25 (19–26)	23 (20–26)	26 (18–26)
Ciprofloxacin (CIP—5 µg)	≥21	16–20	≤15	36 (S)	34	22	26
Sulfamethoxazole /Trimethoprim (SXT—23.75 /1.25 µg)	≥16	11–15	≤10	27 (S)	26 (23–29)	27 (24–32)	R
Streptomycin (S—10 µg)	≥15	12–14	≤11	15 (S)	18 (12–20)	20 (14–22)	20
Tetracycline (TE—30 µg)	≥15	12–14	≤11	25 (S)	26 (18–25)	28 (24–30)	13
Amoxicillin /Clavulanic Acid (Amc—20/10 µg)	≥18	14–17	≤13	27 (S)	16 (18–24)	31 (28–36)	R
Nalidixid Acid (NAL—30 µg)	≥19	14–18	≤13	23 (S)	26	16	R
Sulfisoxazole (G—0.25 µg)	≥17	13–16	≤12	19 (S)	18 (15–23)	27 (24–34)	R
Ceftiofur (XNL—30 µg)	≥21	18–20	≤17	23 (S)	24 (26–31)	28 (27–31)	17 (14–18)

^1^ Diameter of growth in millimeters. ^2^ Experiment control strains.

## Data Availability

The original contributions presented in this study are included in the article/Supplementary Material. Further inquiries can be directed to the corresponding author.

## Supplementary Materials

The following supporting information can be downloaded at: https://www.mdpi.com/article/10.3390/genes16030254/s1, Table S1: Metadata of SSa isolates used in this study. Table provides detailed information on the isolates analyzed, including their geographic origin, source type (clinical, food, environmental, or animal), sequence type (ST), and associated BioProject accession numbers; Table S2: Pangenome analysis of SSa isolates. Table provides comprehensive pangenome data, including the total number of genes identified across 1,954 SSa genomes. It details the distribution of core, accessory and unique genes. Additionally, it includes the nucleotide sequences of all annotated genes.
